# Ruminal bacteria lipopolysaccharides: an immunological and microbial outlook

**DOI:** 10.1186/s40104-022-00692-5

**Published:** 2022-04-14

**Authors:** E. Sarmikasoglou, A. P. Faciola

**Affiliations:** Department of Animal Sciences, University of Florida, Gainesville, FL 32611 USA

**Keywords:** Antagonistic interactions, Inflammation, Microbial communities, Ruminal endotoxin, Symbiosis

## Abstract

Lipopolysaccharides (LPS) are outer membrane components of Gram-negative bacteria made of three regions: the O-antigen; the core oligosaccharide; and a glucosamine disaccharide linked to hydroxy fatty acids, which is named lipid A. The number phosphate groups, and hydroxy fatty acid chains is associated with the immunopotency and the immunomodulatory activity of LPS, where six-acyl chain lipid A with two phosphate groups is found in virulent strains and five- or four-acyl chain lipid A with one phosphate group are found in non-virulent bacteria strains. Ruminal bacteria are predominantly Gram-negative and their LPS have not been thoroughly investigated. In the rumen, LPS is comprised of mixed ruminal LPS. Drawing upon a body of theoretical and applied work, this paper aims to critically review the scientific literature regarding single-species and mixed ruminal bacteria LPS, highlighting the importance of ruminal LPS to the host. Lastly, future research directions are suggested in order to further our understanding of the roles of LPS in the rumen. Possible suggestions for further understanding ruminal LPS include (1) in silico evaluation of major bacteria contributing to ruminal LPS, (2) structural characterization of LPS from prominent ruminal bacteria species, such as ruminal selenomonads and *Megasphaera elsdenii*, and, (3) ruminal epithelial tissue immune response evaluation from single-species and mixed ruminal LPS. In conclusion, this review identifies numerous areas for future research, including setting the basis for future modeling and simulation of host microbiome interactions in ruminants.

## Introduction

The ruminal microbiome is classified as one of the most diverse microbial ecosystems yet reported in the animal kingdom and is composed of bacteria, archaea, protozoa, fungi, and viruses in different abundances [1]. Bacteria are the most abundant and estimated to reach 10^10^–10^11^ bacteria/mL of ruminal fluid [2], thus the manipulation of ruminal bacteria fermentation attracted the interest from researchers in multiple fields such as animal nutrition and rumen microbiology. During ruminal fermentation, lysis of bacteria occurs naturally, thus bacterial toxins constantly interact with the ruminal epithelium [3]. Bacterial toxins are divided into exotoxins, that are secreted from virulent Gram-positive bacteria, and endotoxins, that are excreted mainly after lysis of Gram-negative bacteria [4]. To our knowledge no published data exists showing the presence of ruminal Gram-positive bacteria that secrete exotoxins; however, the presence of endotoxins in the rumen is well defined [5] and consistent with the abundance of Gram-negative bacteria in the rumen microbiome [3].

**Fig. 1:**
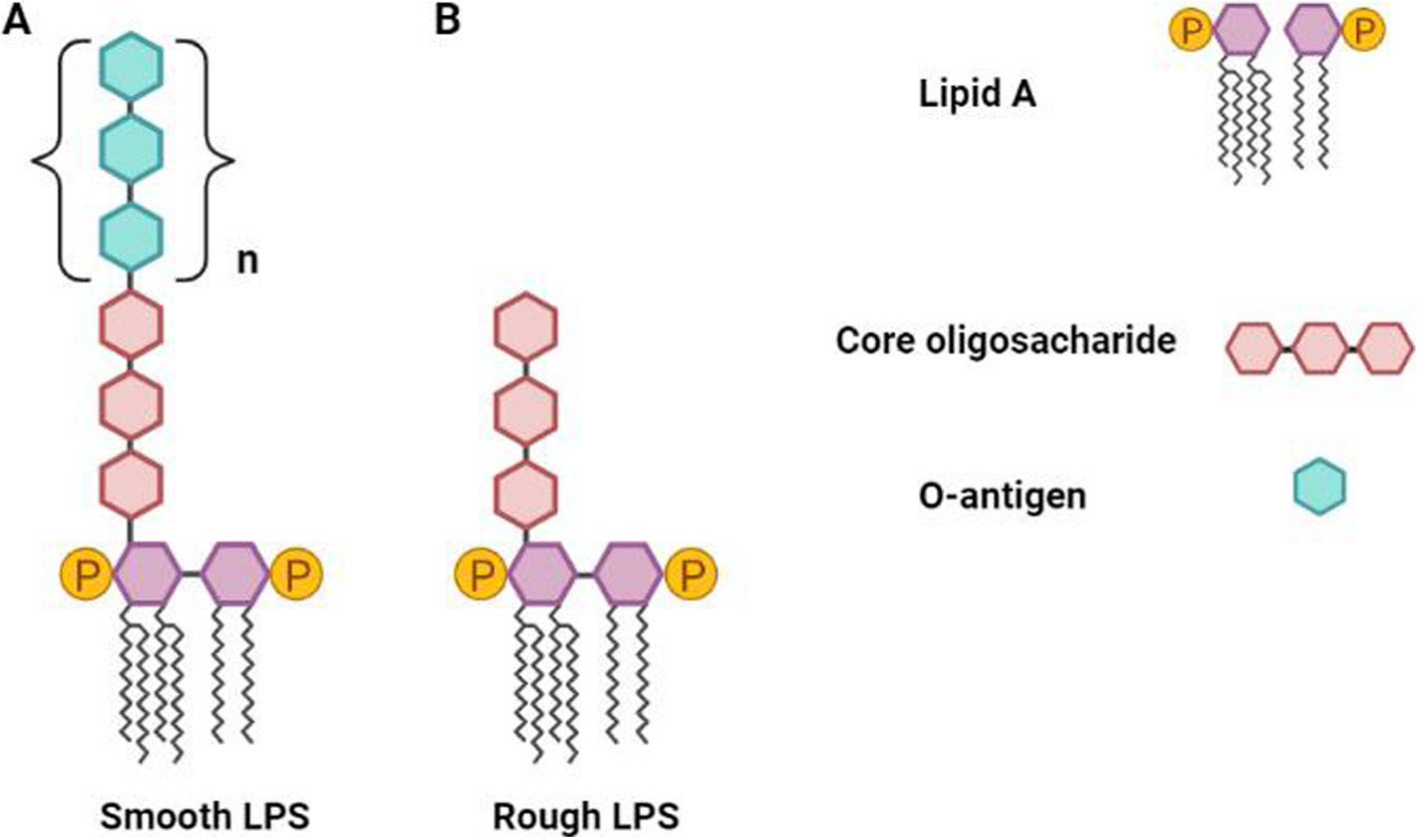
Bacteria that have the smooth LPS phenotype, expressing all three regions of the LPS molecule (**A**) whereas bacteria that have the rough LPS phenotype have lost the expression of the O-antigen chains, expressing only the core oligosaccharide and lipid A (**B**). Created by BioRender.com

Lipopolysaccharides (LPS), are outer membrane components of Gram-negative bacteria that are well-known endotoxins, that, structurally are comprised by three covalently linked regions: the O-antigen, the core oligosaccharide, and the lipid A, whose structure primarily mediates the immunogenicity and the strength of the intracellular signaling to the host [6–8]. In some cases, bacteria express only the core-oligosaccharide and the lipid A, in which such LPS molecules are defined rough LPS (Fig. 1); however, they are mostly artificially designed and are scarce in nature [9]. Among Gram-negative bacteria species there is variation in the structure of the lipid A [10, 11]. Lipid A consists of a glucosamine disaccharide linked with hydroxy fatty acids, also known as fatty acyl chains, which are usually attached to one phosphate group on each carbohydrate [6]. The number of acyl chains on the lipid A are associated with its ability to induce weak or acute immune responses. Hexa-acylated forms usually are the most immunostimulant ones, contrary to the under-acylated forms (penta- or tetra-acylated) that typically result in weak innate immune responses from the host [12, 13].

Lastly, the presence of phosphate groups in the lipid A region significantly increases its immunostimulatory ability [6].

Innate immune responses depend on the germline-encoded receptors called pattern recognition receptors (PRRs) that are located on the host cell membrane. Toll-like receptors (TLRs) are an important family of PRRs which are comprised of approximately 13 members that recognize microorganisms by binding to highly conserved microbial-associated molecular patterns (MAMPs), such as LPS [14]. Microbial recognition from TLRs stimulates the production of cytokines, especially, pro-inflammatory cytokines, interferons, and chemokines that regulate cell growth, activation, and differentiation, contributing to microbial death and clearance by homing of the immune cells to the sites of infection. Each MAMP has a common molecular motif that allows to be recognized by different PRRs; however, independently of their virulence function all microorganisms hold a particular MAMP [15]. Therefore, similarly to virulent bacteria, symbiotic bacteria are recognized as intruders, partially contributing to the host immune response. The symbiotic relationship between the ruminal microbiome and the host maintains homeostasis in the rumen [16]; however, this seems contradictory given that microorganisms induce host immune response, which raises the question: How do ruminants co-habit with their ruminal microbiome?

One widely accepted paradigm suggests that the relationship between the host and microbiome depends on the capacity of the host cell to tolerate the immune stimulation induced by the microbiome such as the activation of TLR4 pathways by LPS [17, 18]. In addition, previous reports suggested that during periods of low ruminal pH, induced by a high grain diet, there is an excessive lysis of virulence strains of *Escherichia coli* that could further trigger inflammatory response in the cow and also reduced feed intake, diarrhea, milk fat depression, liver abscesses, and laminitis [17, 19, 20]. However, regardless of health status, the most predominant phylum in the ruminal fluid is Bacteroidetes, which is composed of species from *Prevotella* and *Bacteroides* genuses that have been suggested to produce LPS with lower endotoxicity compared to LPS produced from virulent bacteria [21].

Consistent with previous findings, a large proportion of ruminal LPS seems to be produced by non-*E. coli* Gram-negative bacteria [22, 23] and most importantly from strains that produce under-acylated isoforms of LPS. These under-acylated isoforms of LPS have been reported to inhibit TLR4 signaling in the human gut thus protecting from the development of gut diseases in humans [24]; however, their functions in bovine rumen health and disease development is still to be elucidated.

In this review, we examined the literature of prevailing mechanisms of host immune tolerance to symbiotic microorganisms in cattle and other hosts, discuss recent findings regarding LPS derived from mixed ruminal microorganisms as well as from pure ruminal bacteria cultures, and finally suggest directions for future research towards understanding the major contributors of ruminal LPS as well as pointing out prominent LPS research on specific ruminal Gram-negative bacteria species.

## Importance of ruminal LPS to host well-being

### Prevailing mechanisms of host immune tolerance to symbiotic microorganisms

The ruminal microbiome are comprised of a dynamic population that form a symbiotic relationship with the host, maintaining homeostasis in the rumen [25]. Proposed homeostasis mechanisms are centered on host-driven tolerance and are associated with the physical barriers of the epithelium (i.e. keratinized outer layer of ruminal epithelium) and mucosa, as well as secretion of antimicrobial peptides [26] or negative feedback loops in nuclear factor kappa-light-chain-enhancer of activated B cells (NF-kB) signaling [27]. Recently, several studies in the human microbiome reported that commensal bacteria have a critical role in the suppression of inflammatory and allergic responses [28] by promoting the expansion of Foxp3^+^ regulatory T cells [29], suppressing tumor necrosis factor alpha (TNF-α) production [30, 31], and supporting gut epithelium health [32], thus indicating that the microbiota could be a driving factor in shaping host immune responses. The immunoinhibitory LPS isoforms that inhibit TLR4 signaling have been suggested as potential mediators of host tolerance in humans [18, 24].

As noted above, in the rumen the continuous fermentation leads to an environment, in which bacteria lyse, and their respective endotoxins are naturally released, creating an environment that is under continual immune-stimulation [3, 33].

In animal studies, host-microbiome interactions are modeled, and studied using potent TLR signaling conditions, such as *E. coli* LPS, to simulate microbial infection [34, 35]. Ruminal bacteria are predominantly Gram-negative with the most predominant phylum in the ruminal fluid to be Bacteroidetes (47–55%), even under subacute ruminal acidosis conditions [22].Bacteroidetes from previous reports seem to express under-acylated LPS isoforms that inhibit the induction of acute immune response to the host [18]. These findings are consistent with preliminary reports suggesting that under-acylated LPS (four or five acyl chains) can be conserved across ruminal LPS [21]. Therefore, specific ruminal LPS is a prominent microbiome-derived molecular mediator of host-tolerance that seems to be considerably different from potent stimulators (such as *E. coli* LPS) that have been frequently used in modeling the host-microbiome interactions in cattle. Lastly, the host-microbiome interactions in cattle should be further examined and modeled based on a more representative LPS such as ruminal LPS.

### Immunomodulatory abilities of LPS expressed from commensal bacteria

The immunopotential of LPS is associated with its specific structure, where agonistic forms induce acute immune response and express hexa-acylated lipid A, while antagonistic forms induce weak immune response and produce under-acylated lipid A [13]. Members of the gut microbiome in humans that are considered commensals, belong to Bacteroidetes phylum, and more specifically in the *Bacteroides* genus [36]. Immunomodulatory properties of *Bacteroides* are known and mostly based on the LPS structure produced by the species of this genus [8]. For instance, *Bacteroides vulgatus* expresses penta-acylated mono-phosphorylated lipid A with profound immune-modulating properties that promote immunological tolerance to mouse models and humans [37, 38]. More specifically, the five acyl chain competes with six acyl chain molecules for host receptor binding and the mono-phosphorylated lipid A results into a neutral charged molecule that mediates interactions with neighboring phosphorylated lipid A. Thus, LPS produced from *Bacteroides vulgatus* exhibits weak agonistic interaction with MD-2/TLR4 and could restore intestinal immune homeostasis after severe inflammation, similar to a colitis challenge in a mouse model [39].

Ruminal bacteria are predominantly Gram-negative, thus bacterial lysis occurs naturally and results in the release of their LPS in the rumen [40, 41]. Under non-healthy conditions, such as ruminal acidosis, free ruminal LPS concentration has been reported to be greater (42,206 EU/mL) compared to healthy cattle (34,179 EU/mL), contributing to systemic inflammation [42]; however, no established association has been shown between the development of ruminal acidosis and ruminal LPS concentration alone [35]. The ruminal microbiome is composed mostly by symbiotic bacteria that seem to produce under-acylated LPS [21]. Additionally, the ruminal microbiota most abundant phylum is Bacteroidetes even under ruminal acidosis conditions [23]. Human Bacteroidetes have been reported to produce under-acylated LPS and exhibit immunomodulatory properties [8], thus similar abilities could be expected from ruminal Bacteroidetes.

Evaluating the expression of pro-inflammatory cytokines (i.e. IL-6, IL-10) in rumen epithelial cells, in the presence of LPS extracted from mixed- and/or mono- cultured ruminal bacteria would give us insights about the potential immunomodulatory abilities of ruminal LPS and how the cow-microbiome interactions should further be modeled. Additionally, future research towards that direction could provide knowledge about the major contributors of ruminal LPS and reveal prominent species for further research.

## Experimental approaches to evaluate the major contributors of ruminal LPS and its properties

Ruminal bacteria contribute to the production of ruminal LPS; however, at the genera and species level this contribution is not well elucidated.

As a starting point, bioinformatics would be a good tool to provide valuable insights for future research. More specifically, the lipid A biosynthesis pathway is conserved across different Gram-negative bacteria and requires nine essential enzymes (LpxA, LpxC, LpxD, LpxH, LpxB, LpxK, WaaA, LpxL, and LpxM) to be functional [43]. First, LpxA (UDP-N-acetylglucosamine acyltransferase) catalyzes the first step of lipid A biosynthesis, which is the transfer of R-3-hydroxymyristic acid from acyl carrier protein to the 3′-hydroxyl group of UDP-GlcNAc. This is followed by the function of the LpxC enzyme, which catalyzes the deacetylation of UDP-3-O-(R-3-hydroxymyristoyl) GlcNAc. Another deacetylation is catalyzed by LpxD, which adds a second R-3-hydroxymyristate chain and make UDP-2,3-diacyl-glucosamine (GlcN). The pyrophosphate linkage of UDP-2,3-diacyl-GlcN, is later cleaved by LpxH to form 2,3-diacyl-GlcN-1-phosphate (lipid X). LpxB condenses UDP-2,3-diacyl-GlcN to generate disaccharide 1-phosphate from lipid X, that is subsequently phosphorylated by LpxK to form the lipid A disaccharide biphosphate (lipid IV). Then, WaaA enzyme catalyzes the incorporation of the two 2-keto-3-deoxy-D-mannooctanoic acid (KDO2) residues, while LpxL incorporates laurate. Lastly, LpxM catalyzes the addition of myristoyl residues to the distal glucosamine unit [44].

With in silico evaluation, we would be able to predict the presence and abundance of each enzyme from the Raetz pathway in selected genera from previous ruminal microbiome studies. This approach would provide knowledge about the major contributors in ruminal LPS production under healthy and non-healthy conditions, such as metabolic disorders associated with septic shock. More specifically, it has been speculated that under heathy conditions, the abundant Gram-negative genera in the rumen would contribute to the synthesis of ruminal LPS [33]; however, this has not yet been experimentally demonstrated. Additionally, similar approaches would further the knowledge on potential correlations of ruminal LPS and disorders in cattle such as ruminal acidosis. Furthermore, in silico approaches are quite useful on giving insights about future research targets, in order to validate these predictions, further experimental approaches should follow. For instance, metagenomic whole genome shotgun sequencing of ruminal contents would allow for the identification of species, even strains that are contributing to LPS production in the rumen, by focusing on the presence of the gene ontology functions (GO) terms associated with LPS biosynthesis, such as the lipid A biosynthetic process (GO: 0009245), the LPS core region biosynthetic process (GO: 0009244) and the LPS biosynthetic process (GO: 0009103). Previous studies have shown that Bacteroidetes species of the human gut microbiome contribute with 79–92.4% of the LPS biosynthesis, contrary to proteobacteria contribution which accounted, on average, for 14% [18]. In the rumen, Bacteroidetes along with Firmicutes are the most dominant phyla. Firmicutes phylum mostly contains Gram-positive bacteria; however, some genera of that phylum, such as *Selenomonas* and *Megasphaera*, have a pseudo-outer membrane and produce LPS and thus, are stained Gram-negative [45]. Therefore, a potential evaluation of the presence of the three main GO terms would provide us information about their contribution to ruminal LPS as well as further insights about potential immunomodulatory ability that ruminal bacteria may exhibit.

Based on the predictive data that would be derived from in silico analysis and metagenomic, whole genome shotgun sequencing, prominent bacteria contributing to ruminal LPS production would be identified even at the species level. Therefore, as the last step in evaluating potential immunomodulatory properties of ruminal LPS would be the structural characterization of LPS, as well as the evaluation of immune responses in ruminal epithelial tissues and animal models, such as mouse and cows, by using LPS derived from specific ruminal bacteria that contribute to ruminal LPS production.

Regarding the structural characterization, the complex nature of LPS molecules require the combination of chemical, spectroscopic, and spectrometric techniques in order to fully define their structures. More specifically, chemical analyses would reveal the nature of LPS (smooth or rough) and its monosaccharide- and fatty acid- composition by applying sodium dodecyl sulfate-polyacrylamide gel electrophoresis followed by silver staining and gas chromatography-mass spectrometry (GC-MS) as fatty acid methyl ester derivative respectively. In addition, to determine the sequence of monosaccharides in the polysaccharidic part (core region and O-antigen), 1D and 2D nuclear magnetic resonance (NMR) spectroscopy would be needed on the deacylated LPS. Lastly, in order to define the structure of the lipid portion (lipid A), matrix-assisted laser desorption/ionization-mass spectrometry (MALDI-MS) would be needed on the hydrolyzed lipid A fraction as previously described in details in our case report [21].

## Prominent bacteria species from previous literature for future LPS structural characterization

### Ruminal selenomonads

S*elenomonas* species have been isolated from a variety of environments such as the rumen [46, 47], the cecum of guinea pigs [48], the human oral cavity [49], and even from ditch water from wetlands [50]. Despite this, selenomonads are usually saccharolytic and the strains isolated from the rumen ferment and utilize lactate, thus their role in the ruminal fermentation is rudimental [5]. Ruminal selenomonads are classified into two strains, *Selenomonas ruminantium* var. *lactilytica* and *Selenomonas ruminantium* var. *ruminantium* that exhibit diametrically opposite functions regarding lactate and glycerol utilization. *S. ruminantium* var. *lactilytica* utilizes lactate and glycerol, whereas *S. ruminantium* var. *ruminantium* ferments glucose and result into lactate production [5, 47].

Lipopolysaccharides extracted from *Selenomonas* spp. isolated from human periodontal pockets exhibited active pyrogenicity, gave a typical biphasic-fever response and produced a positive local Shwartzman reaction [49]. These characteristics correspond to LPS with endotoxic activity quite comparable to LPS produced from well-known virulent species, such as some *E. coli*. As noted earlier, in the rumen, two varieties of *Selenomonas* spp. exist, the *S. ruminantium* var. *ruminantium* and *S. ruminantium* var. *lactilytica* which their respective LPS endotoxicity and structure remains unknown. Considering the high endotoxicity exhibited by *Selenomonas* spp. isolated from the oral cavity it would be meaningful to determine the endotoxin structure of these two strains of ruminal selenomonads by using spectrometric and spectroscopic techniques, such as GC-MS and MALDI-MS. As an expectation, we could hypothesize that both strains of ruminal selenomonads would exhibit hexa-acylated lipid A structures; however, the lipid A structure produced even from the same bacteria species can vary structurally, thus such assumptions need to be validated experimentally before taken under further consideration.

### Megasphaera elsdenii

Another important ruminal microorganism, in addition to *S. ruminantium* var. *lactilytica*, in regards to lactic acid utilization and therefore overall prevention of ruminal lactic acid accumulation, is *Megasphaera elsdenii* [51]. The ability of *M. elsdenii* to utilize lactic acid and produce butyrate, a volatile fatty acid suggested to contribute in oxidative stress reduction in humans [52], as well as, in calf rumen development [53], and well-established probiotic [54]. In commercial probiotics, mostly Gram-positive bacteria species are used and especially from the genus of *Lactobacillus* and *Bifidobacterium* [55]; however, some Gram-negative species with useful properties such as lactate utilization are used as well [56]. However, why does a Gram-negative bacterium such as *M. elsdenii,* does not trigger acute immune responses to the host when used as probiotic?

One previous report evaluated the median lethal doses in mice and in chicken embryos, as well as the minimal dose required to elicit local Shwartzman reaction of the endotoxin derived from *M. elsdenii* isolated from the rumen of cattle [57]*.* Results indicated that the endotoxin of ruminal *M. elsdenii* exhibited low endotoxicity which could imply a structurally under-acylated lipid A. Regarding spectroscopic and spectrometric information about ruminal *M. elsdenii* LPS, the only study, to our knowledge, available in the literature, reports the presence of carbohydrate, protein, lipid, phosphorus, and 2-keto-3-deoxyoctonate [57]. Therefore, a complete structural analysis of the LPS from ruminal *M. elsdenii* would enlighten the reason as to why it does not induce immune response when applied as a probiotic and reveal any potential antagonistic interaction with virulent species LPS in the rumen thus silencing TLR signaling from ruminal epithelium.

## Conclusions

Overall, our literature review identified the scarce number of reports describing some of the bacteria species contributing to LPS production in the rumen, highlighted the potential immunomodulatory properties of ruminal LPS to silence TLR signaling thus inhibiting NF-κB pathway activation, and finally proposed methods that should be followed in order to achieve that. Importantly, the present review is the first to propose experimental approaches towards further understanding the nature of ruminal LPS and its potential immunomodulatory functions by characterizing its complete structure from prominent ruminal bacteria species.

In conclusion, more research is needed to understand host-microbiome interactions, to reevaluate the use of TLR signaling conditions to simulate commensals’ immunostimulant ability and to identify major contributors in specie level on ruminal LPS production under healthy and non-healthy conditions.

## Data Availability

Not applicable.

## Abbreviations

LPS: Lipopolysaccharides
PRRs: Pattern recognition receptors
TLRs: Toll-like receptors
MAMPs: Microbial-associated molecular patterns
NF-kB: Nuclear factor kappa-light-chain-enhancer of activated B cells
TNF-α: Tumor necrosis factor alpha
GC-MS: Gas chromatography-mass spectrometry
NMR: Nuclear magnetic resonance
MALDI-MS: Matrix-assisted laser desorption/ionization-mass spectrometry
